# An explanatory approach to modeling the fleet assignment in the global air transportation system

**DOI:** 10.1007/s13272-022-00622-1

**Published:** 2022-11-24

**Authors:** Markus Kühlen, Klaus Lütjens, Florian Linke, Volker Gollnick

**Affiliations:** 1 Air Transportation Systems, German Aerospace Center (DLR), Blohmstraße 20, 21079 Hamburg, Germany; 2 Institute of Air Transportation Systems, Hamburg University of Technology, Blohmstraße 20, 21079 Hamburg, Germany

**Keywords:** Air transportation system, Fleet assignment, Global air traffic model, Aircraft size, Explanatory modeling

## Abstract

Airlines’ fleet assignment heavily affects the economic and ecological performance of the global air transportation system (ATS). Consequently, it is inevitable to include potential changes of the fleet assignment when modeling and assessing future global ATS scenarios. Therefore, this article presents a novel explanatory approach to modeling the fleet assignment in the global ATS. The presented approach is based on formulating and solving an optimization problem, which describes the fleet assignment in the ATS through a suitable combination of objective function and constraints. While the objective function combines both the airline and the passenger perspective on the fleet assignment, the constraints include additional operational and technological aspects. In comparison to the available global fleet assignment models in the literature, which rely on statistical approaches, the advantages of the presented approach via an optimization problem lie in the overall scenario capability and the consideration of explicit aircraft types instead of simplifying seat categories. To calibrate and validate our model, we use 10 years of historic flight schedule data. The results underline the strengths and weaknesses of the presented approach and indicate potential for future improvement.

## Introduction

Despite the currently ongoing COVID-19 pandemic, the aviation industry is expecting long-term growth of passenger air traffic. Different industry forecasts anticipate reaching pre-pandemic revenue passenger kilometers (RPKs) between 2023 and 2027. For the time span of 2018–2050, the average annual RPK growth rate is estimated to lie between 2.9% and 4.2%; only marginally below pre-pandemic growth estimations. [1–5]

Contrastingly, the aviation industry and political institutions are aiming to reduce emissions and climate impact of air traffic to accomplish the objectives set by the Paris Agreement [6–8]. Hence, the scientific community discusses a plethora of technological, operational, and market-based mitigation measures to reduce the climate impact of aviation. These potential mitigation measures include, among others, alternative jet fuels [9], hybrid-electric aircraft [10], formation flight [11], climate optimized trajectories [12], climate-charged areas [13], and carbon offsetting [14].

However, as with all changes to the ATS, airlines are expected to adopt their operations to defend and strengthen their market positioning, when facing potential mitigation measures. One way of adopting an airline’s operations is by updating the fleet and the fleet assignment, deciding which aircraft type is operated on which route. Together with the finite number of airlines operating in the ATS, these individual airline fleet assignment decisions aggregate into changes of the overall fleet assignment in the global ATS. Because these changes of the overall fleet assignment affect the economic and ecological performance of the global ATS, e.g., by changing the location and quantity of emissions or by changing the number of departures and arrivals at airports, it is, thus, inevitable to include potential changes of the fleet assignment when modeling and assessing future global ATS scenarios.

Available literature on modeling airlines’ fleet assignments can broadly be categorized into approaches for individual airlines, for multiple competing airlines and for the global fleet assignment. Fleet assignment models for individual airlines strive, e.g., for minimal operating cost or maximal operating profit, while considering operational constraints like limited fleet sizes and airport slot restrictions. Methodically, mathematical optimization approaches like linear programming or mixed-integer programming are used. The early work of Abara [15] and Hane et al. [16] focused on optimizing the short-haul fleet assignments of an individual airline given a daily or weekly fixed flight schedule and only a few different aircraft types (up to four and eleven, respectively). More recent work covers larger scale problems as well as additional constraints and interactions. This includes for example integrated flight scheduling and fleet assignment as well as passenger behavior and preferences via supply-demand interactions [17–19].

Fleet assignment models for multiple competing airlines focus on capturing the competitive nature of flight scheduling, fleet assignment and flight frequencies when multiple airlines operate within the same market. Methodically, game theory approaches are applied, in which the competition is portrayed as a non-cooperative game between multiple airlines, each trying to maximize its operational profit or market share. While the early work of Hansen [20] as well as Dobson and Lederer [21] concentrates primarily on the effects of flight scheduling and flight frequency, more recent studies, e.g., by Adler [22], Wei and Hansen [23] as well as Doyme et al. [24], also include the assignment of different aircraft sizes, either by seat categories or explicit aircraft types.

Global fleet assignment models are sparsely available in the literature and mainly focus on statistic modeling approaches.

The Aviation Integrated Model (AIM) [25–27] utilizes a multinomial logit model calibrated with historic flight schedule data to model which size of aircraft is used by airlines on which route. The model variables include route-specific variables like the distance between origin and destination airport, the number of passengers and the passenger load factor on the segment. Additionally, airport-specific variables are included, e.g., the maximum runway length of the origin and destination airport and whether one of these airports is a major hub airport. Nine different aircraft seat categories are used to represent the available aircraft fleet with one representative aircraft type for each category.

Kölker et al. [28] also use seat categories to represent different aircraft types in an aggregated way. Yet, their modeling approach focuses on representing the proportion of flights within a distance and passenger volume band with Gaussian functions for each seat category.

The dependency between aircraft size, flight distance, and passenger volume on a route is also employed by the Future Air Traffic Estimator (FATE) model [29]. Unfortunately, a more detailed description of the model methodology is not published.

Similarly, the publications about how the fleet assignment in the global ATS is modeled within the Aviation environmental Portfolio Management Tool (APMT) [30, 31] and the Aviation Emissions and evaluation of Reduction Options Modeling System (AERO-MS) [32, 33] are sparse. For the APMT only aims and conceptual model architectures are published, stating the model shall be able to describe implications of technological and operational changes for the fleet assignment. In AERO-MS, the mix of aircraft to operate on a route is supposed to be determined in dependency of the operating cost of different aircraft sizes and range categories. Though, no methodology is given on how this is achieved.

In this article, we present a new explanatory approach to modeling the fleet assignment in the global ATS. Similar to the previously discussed approaches for modeling the fleet assignment of an individual airline, our approach is based on formulating and solving an optimization problem. However, rather than optimizing the fleet assignment and flight scheduling of an individual airline, we focus on capturing the dynamics of the global, cross-airline fleet assignment in the ATS. Therefore, we apply a different objective function and a different set of constraints. Our objective function includes the airlines’ and passengers’ perspectives, via direct operating cost (DOC), schedule delay and travel time. Our constraints represent logical, operational, and technological aspects of the fleet assignment, like limited fleet availability, airport capacities, and payload-range capabilities of the different aircraft types. Effects of flight scheduling and airline competition, which are key elements in fleet assignment models for individual airlines and multiple competing airlines, are substitutionally included into our optimization problem via a utilization model and market concentration constraints. In doing so, we achieve a suitable level of detail for modeling the global ATS and ease the computational requirements of our optimization model; leading to computation times of less than ten minutes for the yearly fleet assignment of the global ATS. To calibrate and validate the overall model, we use ten years of historic flight schedule data. In contrast to the statistical modeling approaches for the global fleet assignment, which are available in the literature, our new explanatory approach via an optimization problem has the two main advantages of allowing diverse scenario analyses and including explicit aircraft types instead of aircraft seat categories.

The remainder of this article is arranged as follows: Section 2 describes in detail the methodology of this new explanatory approach to modeling the fleet assignment in the global ATS. Section 3 presents exemplary results achieved with this new methodology. In Sect. 4, these results are discussed. Section 5 concludes this article and gives an outlook on future research.

## Methodology

This section presents first the data sets used for this study; second, the developed overall model architecture; and third, the underlying submodels. Last, the mathematical formulation of the model as an optimization problem is introduced.

### Data sets

The Sabre Market Intelligence database provides global historic flight schedule data for the years 2010–2019 [34]. This historic flight schedule data yield information on the global route network, on flight frequencies and seat capacities for each origin-destination pair as well as information on aircraft utilization and available seats per aircraft type.

The Cirium Fleets Analyzer database provides information about the global aircraft fleet for the years 2010–2019 [35]. The fleet data includes, among others, the number of available units per aircraft type as well as weight and utilization information for each aircraft unit. For each year, the fleet is assumed to be constant; with the fleet on June 30 being representative for the whole year.

The available overall data sets regarding route networks and aircraft fleets are split into two subsets. The first, spanning the years 2010–2016, is used for model calibration and submodel development. The second one, spanning the years 2017–2019, is used for model validation.

Aircraft type performance data are taken from the Aircraft Characteristic Manuals (ACMs) provided by the aircraft manufacturers [36, 37] and from the type certificate data sheets [38]. These performance data include payload-range diagrams, maximum seating capacities as well as required takeoff and landing field length information.

Data regarding aircraft-type-specific fuel consumption is derived from EUROCONTROL’s Base of Aircraft Data (BADA) data sets [39].

The required airport-specific information consists of IATA codes, geographic coordinates, number of runways, maximum runway lengths, and the runway configurations. The information is collected and aggregated from various publicly available data sources, e.g., Wikipedia and Google’s satellite imagery.

While most of the data mentioned above would be available for a wide variety of different aircraft types, this study focuses on a selected group of relevant passenger aircraft types, which are considered to be representative for the global ATS. The selected 35 Airbus and Boeing aircraft types cover 63% of global departures and 91% of the global available seat kilometers (ASKs) in the historic flight schedule data. A complete list of the selected aircraft types is given on the abscissae of Figs. 2 and 3.

### Overall model structure

**Fig. 1:**
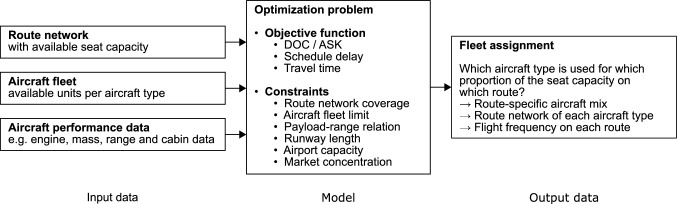
Schematic representation of the overall model architecture

The overall model structure is displayed in Fig. 1. The required input data include the route network, the aircraft fleet and the aircraft performance data. The route network consists of all origin–destination pairs and the offered seat capacity for each pair. The aircraft fleet is characterized by the number of available units per aircraft type. The aircraft performance data, which is required to correctly model the individual operational characteristics of each aircraft type, includes, among others, engine, mass, range, and cabin data.

The fleet assignment model for the global ATS is based on the formulation and solving of an optimization problem. The aim of this optimization problem is to determine which proportion of the offered seat capacity on each route is provided by which aircraft type.

The objective function of the optimization problem includes both the airline and the passenger perspective, representing the cost and revenue side of aircraft operation, respectively. The costs are included into the objective function via the DOC per ASK. The revenues are modeled indirectly via the schedule delay and the travel time, both affecting the passenger acceptance of a fleet assignment and therefore the revenue on a route. The weighting between the different aspects of the objective function is determined during the model calibration process, minimizing the differences between calibration data and model results.

The constraints of the optimization problem consist of logical, operational and technological constraints. Valid solutions of the optimization problem are required to cover the entire seat capacity of each route. Additionally, the limited availability of each aircraft type in the overall aircraft fleet must be considered. Furthermore, the individual relation between payload and range must be met for each aircraft type. From an infrastructure perspective, the available runway length and runway capacity at each airport must be considered. An additional market concentration constraint takes account of the existence of different airlines with individual fleets and route networks.

The solution of the optimization problem results in the primary output of the model: the fleet assignment in the global ATS, answering which aircraft type is used to offer which proportion of the seat capacity on which route. This primary output then leads to additional outputs like the route-specific aircraft mix, the route network of each individual aircraft type and the flight frequency on each route.

### Submodels

Several submodels are used as part of the overall model structure. In the following these submodels are introduced and briefly described.

#### Direct operating cost

The DOC submodel uses the TU Berlin method by J. Thorbeck; also known as the CeRAS DOC model. The TU Berlin method describes the DOC as the sum of the five cost components fuel, maintenance, fees, crew and capital. For each component the method provides an analytic formula for calculating the monetary values in 2010 euro values. [40, 41]

#### Fuel consumption

For the calculation of flight-specific fuel consumption DLR’s Trajectory Calculation Module (TCM) is applied [42, 43]. This tool performs a three-degree-of-freedom trajectory simulation with simplified equations of motion, also known as Total Energy Model, and employs the aircraft performance models provided in the BADA (model family 4) [44]. With TCM, we create a database of *reduced* flight profiles by simulating aircraft trajectories for all discrete combinations of flight distance (in steps of 100 NM) and load factors (with a higher resolution around usual load factors between 0.75 and 0.85) for all aircraft models available in BADA 4. For the simulation standard climb and descent procedures are assumed and corresponding speed schedules are used. During cruise, long-range cruise speed is applied and step climbs are modeled to approximate operationally realistic cruise profiles. We compress the trajectory data (= *reduction*) by saving the relevant aircraft state parameters (i.e., flown distance, time, altitude, fuel flow) at significant profile vertices only, i.e., transitions between flight phases, into the database, as also, e.g., done by Linke et al. [45, 46]. Simple functions can be used to synthesize profiles from the database for various purposes later assuming linearity of aircraft state parameters within individual flight phases. By integrating the fuel flow data over time, we calculate the amount of required mission fuel for all combinations of flight distance and load factor. Based on these fuel values, we model the fuel consumption of operating aircraft type *t* on the route (*o*, *d*) between origin airport *o* and destination airport *d* via the quadratic expression given by Eq. (1); with the fuel mass ${m}_{o, d, t}^{fuel}$, the flight distance $d_{o, d}$, the load factor ${lf}_{o, d, t}$ and the aircraft-type-specific regression coefficients $a_{i, t}$. To incorporate lateral ground track extensions due to routing inefficiencies, we use Eq. (2) for calculating the flight distance $d_{o, d}$ based on the great circle distance ${gcd}_{o, d}$ between origin and destination airport [26].

1 $$\begin{aligned} {m}_{o, d, t}^{fuel}&= a_{1, t} \cdot d_{o, d}^2 + a_{2, t} \cdot d_{o, d} + a_{3, t} \cdot d_{o, d} \cdot {lf}_{o, d, t} + a_{4,t}, \end{aligned}$$

2 $$\begin{aligned} {d}_{o, d}&= 40.74 \, \text {km} + 1.029 \cdot gcd_{o, d}. \end{aligned}$$

#### Utilization

The utilization submodel describes the yearly service and transport performance of an aircraft unit. The block time ${bt}_{o, d, t}$ for servicing a route (*o*, *d*) with aircraft type *t* is calculated in dependency of the great circle distance ${gcd}_{o, d}$ between origin and destination airport and the typical cruise Mach number of the used aircraft type ${Ma}_{t}^{cr}$. According to the ACMs [36, 37] and literature studies [47, 48], the critical path for full service turnarounds is defined by passenger deplaning and boarding as well as catering and cleaning or refueling. Thus, the turnaround time ${tat}_{o, d, t}$ between two flights when servicing a route (*o*, *d*) with aircraft type *t* is modeled in dependency of the number of available passenger seats per flight $p_{o, d, t}$ and the block time ${bt}_{o, d, t}$. The utilization rate ${utr}_{o, d, t}$, which describes the percentage of time used for performing flight operations when servicing a route (*o*, *d*) with aircraft type *t*, including both flights and turnarounds, is modeled in dependency of the block time ${BT}_{o, d, t}$ and the aircraft age $age_{t}$.

3 $$\begin{aligned} {bt}_{o, d, t}&= \frac{a_5}{\left( {Ma}_{t}^{cr}\right) ^{a_6}} \cdot {gcd}_{o, d} \nonumber \\&\quad - \exp \left( -a_7 \cdot {gcd}_{o, d} \right) + a_8 + 1, \end{aligned}$$

4 $$\begin{aligned} {tat}_{o, d, t}&= a_9 \cdot p_{o, d, t} + a_{10} \cdot {bt}_{o, d, t} + a_{11}, \end{aligned}$$

5 $$\begin{aligned} {utr}_{o, d, t}&= 1 - \exp \left( -a_{12} \cdot {bt}_{o, d, t}^{a_{13}} \right) - a_{14} \cdot age_{t}. \end{aligned}$$

The coefficients $a_5$–$a_{14}$ are derived from historic flight schedule and aircraft utilization data. Thus, the utilization submodel implicitly includes a variety of effects on aircraft utilization, which are not explicitly modeled, e.g., effects of flight routing on block time, schedule buffer on turn around time, and night curfews and airlines’ aircraft flow balance on utilization rate.

#### Payload-range relation

The payload-range relation of each aircraft type is directly derived from the ACMs. For each aircraft type a representative weight variant is selected and the boundaries of the payload-range diagram are modeled as straight lines.

#### Required field length

The required takeoff and landing field lengths, ${tofl}_{o, d, t, p}^{req}$ and ${lfl}_{o, d, t, p}^{req}$ when operating aircraft type *t* with *p* passengers on route (*o*, *d*) are modeled with the analytic formulas given in Eqs. (6) and (7); with the takeoff mass ${tom}_{o, d, t, p}$, the landing mass ${lm}_{o, d, t, p}$ and the origin and destination airport pressure altitudes ${pa}_{o}$ and ${pa}_{d}$. The aircraft-type-specific coefficients $a_{15, t}$ through $a_{24, t}$ are derived from the takeoff and landing performance data in the ACMs.

6 $$\begin{aligned}&\begin{aligned}&{}{tofl}_{o, d, t, p}^{req} = a_{15, t} \cdot \exp (a_{16, t} \cdot {tom}_{o, d, t, p} \\&\quad + a_{17, t} \cdot {tom}_{o, d, t, p} \cdot {pa}_{o} \\&\quad + a_{18, t} \cdot {tom}_{o, d, t, p} \cdot {pa}_{o}^2 ) + a_{19, t}, \end{aligned} \end{aligned}$$

7 $$\begin{aligned}&\begin{aligned}&{}{lfl}_{o, d, t, p}^{req} = \left( a_{20, t} \right. \\&\quad \left. + a_{21, t} \cdot {lm}_{o, d, t, p} \right) \cdot \exp \left( a_{22, t} \cdot {pa}_{d}\right) \\&\quad + a_{23, t} \cdot {lm}_{o, d, t, p} + a_{24, t} \cdot {{lm}_{o, d, t, p}}^2. \end{aligned} \end{aligned}$$

#### Airport capacity

The methodology for determining the airport capacities is adopted from Gelhausen et al. [49]. Within this methodology, the runway capacity is assumed to be limiting for the airport capacity. Therefore, the historic flight schedule data are used for determining the 95%-runway capacity per hour as well as the operating hours of each airport. Then, the airports are grouped based on the number of available runways and their configuration to determine the maximum 95%-runway capacity per hour of each group. Finally, the yearly runway capacity of each airport is calculated by multiplying the 95%-runway capacity per hour of the airport’s group with the individual yearly operating hours of each airport.

To account for the fact that only a selected group of representative aircraft types are included within this study, the calculated capacity of each airport is additionally scaled with an airport-specific factor. This scaling factor is determined by the ratio of departures and arrivals of the selected group of aircraft types in comparison to the total number of departures and arrivals at each airport.

#### Schedule delay

In general, schedule delay is defined as the difference between the favored departure time of the passenger and the offered departure time in the flight schedule. However, within this study, we use the concept of schedule delay for quantifying the attractiveness of an offered flight frequency from a passenger perspective. Consequently, we apply an aggregated schedule delay approach, adopted from Brueckner and Flores-Fillol [50], where passengers are assumed to care about the offered flight frequency rather than the departure times of individual flights. Additionally and in accordance with Presto et al. [51], we assume a time-wise constant demand and a time-wise equidistant flight schedule. Hence, the average schedule delay ${asd}_{o, d, t}$ of passengers on a route (*o*, *d*) served by $f_{o, d, t}$ flights of aircraft type *t* in a time interval ${\Delta t}_{o, d}$ can be calculated with Eq. (8). For the total schedule delay ${tsd}_{o, d, t}$ of all offered seats on a route follows Eq. (9); using $tsd = asd \cdot capa$ and $capa = f \cdot p$, with *p* being the available seats per flight. As shown by Eq. (9), the total schedule delay ${tsd}_{o, d, t}$ only depends on the considered time interval ${\Delta t}_{o, d}$ and the available seats per flight $p_{o, d, t}$, but not on the overall available seat capacity $capa_{o, d}$ of the route.

8 $$\begin{aligned} {asd}_{o, d, t}&= \frac{1}{4} \cdot \frac{{\Delta t}_{o, d}}{f_{o, d, t} }, \end{aligned}$$

9 $$\begin{aligned} {tsd}_{o, d, t}&= \frac{1}{4} \cdot {\Delta t}_{o, d} \cdot p_{o, d, t}. \end{aligned}$$

While the made assumptions may only be fully accurate for some passengers, e.g., business travelers with unpredictable travel times and flexible tickets, and some routes, e.g., high-frequency routes at airline hubs, the resulting Eqs. (8) and (9) capture the overall and general dynamics between aircraft size, flight frequency, and schedule delay. Thus, we see the approaches by Brueckner and Flores-Fillol [50] and Presto et al. [51] as suitable approximations, given the global and aggregated scope of our model. For using the calculated total schedule delay ${tsd}_{o, d, t}$ within the objective function of the optimization problem, a monetary factor, which is determined during the model calibration, is applied to transform the schedule delay value into a monetary value.

#### Travel time

The travel time evaluation for the objective function is based on the relative comparison of block times of different feasible fleet assignments. With Eq. (10), the additional required block time ${\Delta bt}_{o, d, t}$ for serving the route (*o*, *d*) with aircraft type *t* in comparison to serving it with the fastest available aircraft type ${\overline{t}}$ out of the set of feasible aircraft types $T_{o, d}$ is calculated. By analogy with the schedule delay, the difference in block time ${\Delta bt}_{o, d, t}$ is transferred into a monetary value by applying a monetary factor determined during the calibration process.

10 $$\begin{aligned} {\Delta bt}_{o, d, t} = {bt}_{o, d, t} - \min _{{\overline{t}} \in T_{o, d}} {bt}_{o, d, {\overline{t}}}. \end{aligned}$$

#### Market concentration

The historic flight schedule data indicate a negative correlation between the offered seat capacity on a route and the market concentration of the used aircraft types. This correlation is assumed to be caused by the existence of different airlines with individual fleets and route networks in the global ATS. Thus, this effect is inherently not included in the presented methodology due to the used cross-airline approach. Therefore, the market concentration submodel calculates an upper limit $\overline{{hhi}_{o, d}^{t}}$ for the aircraft type market concentration of individual routes by linking the offered seat capacity on a route $capa_{o, d}$ to the Herfindahl–Hirschman market concentration index (HHI), see Eq. (11). The regression coefficients $a_{25}$ and $a_{26}$ are derived from the historic flight schedule data.

11 $$\begin{aligned} \begin{aligned}&{}\overline{{hhi}_{o, d}^{t}} = a_{25} + \exp \left( - a_{26} \cdot {capa}_{o, d} + \ln \left( 1 - a_{25} \right) \right) . \end{aligned} \end{aligned}$$

### Optimization problem

**Table 1:** Notations for the formulation of the optimization problem

**Sets and parameters**	
*A*	Set of airports in route network; indexed by *a*
*OD*	set of routes in route network; indexed by tuple (*o*, *d*) with origin airport *o* and destination airport *d*
$OD_t$	Subset of routes, which aircraft type *t* can serve
*T*	Set of aircraft types in fleet; indexed by *t*
$T_{o, d}$	Subset of aircraft types, which can serve route (*o*, *d*)
*ODT*	Set of route and aircraft type combinations; indexed by tuple (*o*, *d*, *t*) with origin airport *o*, destination airport *d* and aircraft type *t*
${ARR}_{a}$, ${DEP}_{a}$	Subsets of route and aircraft type combinations, which arrive at and departure from airport *a*, respectively
${c}_{o, d, t}$	Cost to provide the required seat capacity ${capa}_{o, d}$ for route (*o*, *d*) when operating aircraft type *t*
${capa}_{o, d}$	Required seat capacity of route (*o*, *d*)
${u}_{o, d, t}$	Required number of units of aircraft type *t* to provide the required seat capacity ${capa}_{o, d}$
${u}_{t}^{avail}$	Available number of units of aircraft type *t* in the fleet
${f}_{o, d, t}$	Required number of flights of aircraft type *t* to provide the required seat capacity ${capa}_{o, d}$
${f}_a^{avail}$	Available number of aircraft movements at airport *a*; see Sect. 2.3.6
${hhi}_{o, d}^{t}$	HHI of the aircraft types operated on route (*o*, *d*)
$\overline{{hhi}_{o, d}^{t}}$	Upper limit of the HHI of the aircraft types operated on route (*o*, *d*); see Sect. 2.3.9
${P}_{o, d, t}$	Set of the feasible number of offered passenger seats per flight with aircraft type *t* on route (*o*, *d*)
${p}_{o, d, t}$	Number of offered passenger seats per flight with aircraft type *t* on route (*o*, *d*)
$p_{max, t}$	Maximum number of offered passenger seats per flight in an average cabin layout of aircraft type *t*
$r_{t, p}$	Range of aircraft type *t* when operating with *p* passengers; calculated via the payload-range diagram; see Sect. 2.3.4
$d_{o, d}$	Flight distance of route (*o*, *d*)
${tofl}_{o, d, t, p}^{req}$, ${lfl}_{o, d, t, p}^{req}$	Required takeoff and landing field length when operating aircraft type *t* with *p* passengers on route (*o*, *d*); see Sect. 2.3.5
${rwl}_{a}^{avail}$	Available runway length at airport *a*
${doc}_{o, d, t}$	Direct operating cost to provide the required seat capacity ${capa}_{o, d}$ for route (*o*, *d*) with aircraft type *t*; see Sects. 2.3.1– 2.3.3
${tsd}_{o, d, t}$	Total schedule delay of all passengers when operating aircraft type *t* on route (*o*, *d*); see Sect. 2.3.7
${\Delta bt}_{o, d, t}$	Additional block time required to operate aircraft type *t* on route (*o*, *d*) in comparison to operating it with the fastest available aircraft type; see Sect. 2.3.8
${bt}_{o, d, t}$, ${tat}_{o, d, t}$, ${utr}_{o, d, t}$	Block time, turn around time and utilization rate when operating aircraft type *t* on route (*o*, *d*); see Sect. 2.3.3
${\Delta t}^{rn}$	Time period of the route network (e.g., hours per year)
$\beta _1$, $\beta _2$	Calibrated weighting parameters for the cost calculation

**Decision variables**	
$x_{o, d, t} \in \left[ 0, 1 \right]$	Share of the required seat capacity ${capa}_{o, d}$ on route (*o*, *d*), which is served by aircraft type *t*

The modeled optimization problem is described by the objective function and constraints in Eqs. (12)–(16), the parameter calculations in Eqs. (17)–(21), the set definitions in Eqs. (22)–(27) and the notation explanation in Tab. 1.

The aim of the optimization model is to determine a global fleet assignment, which minimizes the cost of serving a route and demand network with an aircraft fleet, Eq. (12). The fleet assignment is given by the decision variables $x_{o, d, t}$, which describe which share of the required seat capacity ${capa}_{o, d}$ on a route between origin airport *o* and destination airport *d* is operated by aircraft type *t*.

To describe a valid solution of the optimization problem, the fleet assignment must meet the following constraints: For each route in the global route network, given by the directed tuple of origin and destination airport (*o*, *d*), the fleet assignment must cover at least the required seat capacity, Eq. (13). For each aircraft type *t*, the fleet assignment must respect the number of available aircraft units in the fleet ${avail\_u}_{t}$, Eq. (14). For each airport *a*, the sum of all departures and all arrivals must be less than or equal to the runway capacity at the given airport, Eq. (15). For each route (*o*, *d*), the market concentration of the used aircraft types operated on this route, given by the HHI ${hhi}_{o, d}^{t}$, must be less than or equal to an upper HHI limit $\overline{{hhi}_{o, d}^{t}}$, Eq. (16). This upper HHI limit is calculated for each route with the regression model described by Eq. (11) in Sect. 2.3.9 based on the required seat capacity ${capa}_{o, d}$.

Equations (17) and (18) ensure that the aircraft types are evaluated with the most cost-efficient, yet operationally feasible number of offered passenger seats per flight $p_{o, d, t}$. First, the number of offered passenger seats $p_{o, d, t}$ must be a strict positive integer less than or equal to the number of seats of an average cabin layout $p_{max, t}$ of the aircraft type *t*. Second, the number of offered passenger seats per flight $p_{o, d, t}$ of aircraft type *t* on route (*o*, *d*) must allow an operating range $r_{t, p}$ equal to or greater than the flight distance $d_{o, d}$ of the route. Third, $p_{o, d, t}$ must allow required takeoff and landing field lengths, ${tofl}_{t, p, o}^{req}$ and ${lfl}_{t, p, d}^{req}$, equal to or less than the available runway lengths at the origin and destination airports, ${rwl}_{o}^{avail}$ and ${rwl}_{d}^{avail}$, respectively. Last, the maximum number of offered passenger seats, which meets the previously defined criteria, is selected for each combination of route and aircraft type, Eq. (18), as this is, given the objective function, the most cost-efficient operating point.

The cost for using aircraft type *t* to provide the required seat capacity $capa_{o, d}$ for the route (*o*, *d*) is the weighted sum of the DOC ${doc}_{o, d, t}$, the total schedule delay $tsd_{o, d, t}$ and the additional block time ${\Delta bt}_{o, d, t}$ of the aircraft type and route combination, Eq. (19). The total schedule delay $tsd_{o, d, t}$ and the additional block time ${\Delta bt}_{o, d, t}$ are calculated as previously described by Eqs. (9) and (10) in Sects. 2.3.7 and 2.3.8. The weighting parameters $\beta _1$ and $\beta _2$ are determined during the model calibration. To calculate the required number of flights $f_{o, d, t}$ with aircraft type *t* to provide the required seat capacity $capa_{o, d}$ for the route (*o*, *d*), the required seat capacity ${capa}_{o, d}$ is divided by the number of offered passenger seats per flight $p_{o, d, t}$, Eq. (20). The required number of units $u_{o, d, t}$ of aircraft type *t* to provide the required seat capacity $capa_{o, d}$ for the route (*o*, *d*) is calculated by dividing the required operating time for the route by the available operating time per aircraft unit, Eq. (21).

**Objective function and constraints:**

12 $$\begin{aligned} \min&\quad \sum _{(o, d, t) \in {ODT}}^{} c_{o, d, t} \cdot x_{o, d, t}, \end{aligned}$$

13 $$\begin{aligned} s.t.&\quad \sum _{t\in T_{o, d}}^{} x_{o, d, t} \ge 1&\forall (o, d) \in {OD}, \end{aligned}$$

14 $$\begin{aligned}&\quad \sum _{(o, d) \in {OD_t}}^{} u_{o, d, t} \cdot x_{o, d, t} \le {u}_{t}^{avail}&\forall t \in T, \end{aligned}$$

15 $$\begin{aligned}&\quad \sum _{(o, d, t) \in ARR_{a}}^{} f_{o, d, t} \cdot x_{o, d, t} + \sum _{(o, d, t) \in DEP_{a}}^{} f_{o, d, t} \cdot x_{o, d, t} \le {f}_a^{avail}&\forall a \in A, \end{aligned}$$

16 $$\begin{aligned}&\quad \sum _{t \in T_{o, d}}^{} {x_{o, d, t}}^{2} = {hhi}_{o, d}^{t} \le \overline{{hhi}_{o, d}^{t}}&\forall (o, d) \in {OD}. \end{aligned}$$

**Parameters:**

17 $$\begin{aligned}&\quad P_{o, d, t} = \Big \{p \in {\mathbb {N}}_{\le p_{max, t}}^{+} \mathrel{\Big|} \big( r_{t, p} \ge d_{o, d} \big) \wedge \big( {tofl}_{o, d, t, p}^{req} \le {rwl}_{o}^{avail} \big) \wedge \big( {lfl}_{o, d, t, p}^{req} \le {rwl}_{d}^{avail} \big) \Big \}&\forall (o, d, t) \in OD \times T, \end{aligned}$$

18 $$\begin{aligned}&\quad {p}_{o, d, t} = \max \left( P_{o, d, t} \right)&\forall (o, d, t) \in ODT, \end{aligned}$$

19 $$\begin{aligned}&\quad c_{o, d, t} = {doc}_{o, d, t} + \beta _{1} \cdot {tsd}_{o, d, t} + \beta _{2} \cdot \Delta {bt}_{o, d, t}&\forall (o, d, t) \in ODT, \end{aligned}$$

20 $$\begin{aligned}&\quad f_{o, d, t} = {capa}_{o, d} \, / \, p_{o, d, t}&\forall (o, d, t) \in {ODT}, \end{aligned}$$

21 $$\begin{aligned}&\quad u_{o, d, t} = f_{o, d, t} \cdot \left( {bt}_{o, d, t} + {tat}_{o, d, t} \right) \, / \, \left( {\Delta t}^{rn} \cdot {utr}_{o, d, t} \right)&\forall (o, d, t) \in {ODT}. \end{aligned}$$

**Sets:**

22 $$\begin{aligned}&\quad {OD} = \left\{ (o, d) \in A \times A \mid o \ne d \wedge {capa}_{o, d} > 0 \right\} , \end{aligned}$$

23 $$\begin{aligned}&\quad {ODT} = \left\{ (o, d, t) \in OD \times T \mid {P}_{o, d, t} \ne \emptyset \right\} , \end{aligned}$$

24 $$\begin{aligned}&\quad {OD}_t = OD \setminus \left\{ (o, d) \in OD \mid P_{o, d, t} = \emptyset \right\}&\forall t \in T, \end{aligned}$$

25 $$\begin{aligned}&\quad T_{o, d} = T \setminus \left\{ t \in T \mid P_{o, d, t} = \emptyset \right\}&\forall (o, d) \in OD, \end{aligned}$$

26 $$\begin{aligned}&\quad ARR_{a} = \left\{ (o, d, t) \in ODT \mid d = a \right\}&\forall a \in A, \end{aligned}$$

27 $$\begin{aligned}&\quad DEP_{a} = \left\{ (o, d, t) \in ODT \mid o = a \right\}&\forall a \in A. \end{aligned}$$

Equations (22)–(27) define sets and subsets of routes, aircraft types and their respective combinations for formulating the optimization problem. The set of routes *OD* is described by the directed tuple (*o*, *d*) of origin airport *o* and destination airport *d*. Of all possible combinations of airports, only those origin and destination airport tuples (*o*, *d*) with strict positive flight distance and strict positive offered seat capacity ${capa}_{o, d}$ are part of the set of routes *OD*, Eq. (22). The set *ODT* includes all tuples (*o*, *d*, *t*) of potential combinations of routes and aircraft types. Only those routes and aircraft type combinations are considered, for which the set of the possible number of offered passenger seats $P_{o, d, t}$ is not empty, Eq. (23). This excludes all combinations, where the range, the takeoff or the landing performance of an aircraft type is not sufficient for serving a route. The subsets $OD_{t}$ include all route tuples (*o*, *d*), which can be served by the aircraft type *t*, Eq. (24). The subsets $T_{od}$ include all aircraft types *t*, which can serve the route (*o*, *d*), Eq. (25). The subsets ${ARR}_{a}$ and ${DEP}_{a}$ include all tuples of potential route and aircraft type combinations, which arrive at and departure from airport *a*, respectively, Eqs. (26) and (27).

The previously presented formulation of the optimization problem leads to a linear program with both linear constraints, Eqs. (13)–(15), and quadratic constraints, Eq. (16). The size of the optimization problem depends primarily on the number of routes and airports in the route network as well as the number of aircraft types in the fleet. For the analyses presented in this paper, the size of the optimization problem varies between approximately 30 and 50 thousand constraints, 0.5 and 1.5 million variables as well as  2.5 and 6.0 million non-zero variable-constraint coefficients. We use *Gurobi Optimizer* [52] to solve the optimization problem. The solver transforms the presented formulation of the optimization problem into a second-order cone problem formulation and applies its barrier method for solving. On a regular laptop with a 9th generation Intel i7 processor (6 physical cores, 2.6 GHz) and 32 GB RAM, solving the optimization problem takes approximately 100–550 seconds.

Currently, the optimization problem does not include any constraints for the flow of aircraft in the route network, e.g., requiring the number of departures and arrivals being equal per aircraft type and airport. This is because the input data for the route network includes significant sources and sinks regarding the offered seat capacity per airport. Therefore, including constraints on the flow of aircraft would require either manipulating the input data or adding the option of additional empty flights to the optimization problem. Both approaches are considered out of scope for this study.

Moreover, the optimization problem also does not require the number of flights on a route to be integer, see Eq. (20). We rate this as a suitable approximation as, first, the aim of the presented methodology is to model the yearly fleet assignment in the global ATS and the majority of flights and ASKs are offered on high frequency routes. For example, combinations of aircraft types and routes with 20 (100) or more flights per year contributed 99% (90%) of the ASKs in the route network input data for 2010. Thus, we expect the modeling error of allowing non-integer flight frequency solutions to be insignificant on a global scale. Second, the alternative of requiring the number of flights on a route to be integer would transform the optimization problem from a linear program into a mixed-integer program, which would significantly increase the computational complexity of solving the optimization problem.

## Results

Using the previously described methodology and data sets to reproduce the fleet assignment in the global ATS for the years 2010–2019 leads to the following results.

### Fleet assignment per aircraft type

**Fig. 2:**
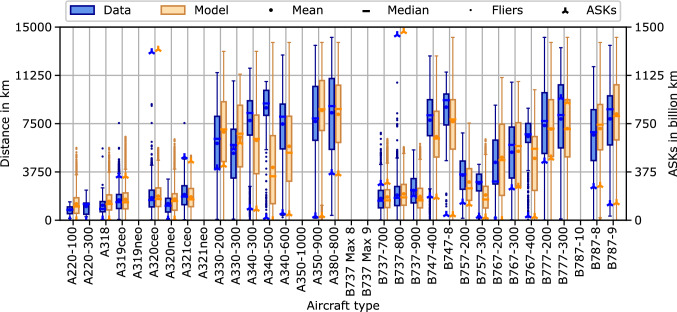
Comparison of aircraft type distance distributions between historic flight schedule data and model results for the year 2016

**Fig. 3:**
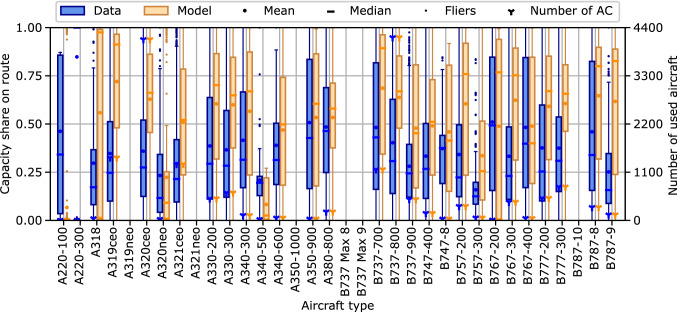
Comparison of aircraft type capacity share distributions between historic flight schedule data and model results for the year 2016

Figure 2 visualizes the distance distributions of individual aircraft types in the 2016 fleet assignment, comparing historic flight schedule data with model results. Additionally, the ASKs for each aircraft type are plotted on the second ordinate and displayed with an asterisk symbol. The results show closer resemblance for narrow body aircraft than for wide body aircraft. Furthermore, aircraft types with lower ASKs, e.g., A340-500, B757-300 and B767-400, tend to show larger differences than aircraft types with higher ASKs. For the A220-300, no results were obtained from the model as the entry into service of the aircraft type took place in the second half of the year, preventing an inclusion in the representative aircraft fleet input data from end of June the same year.

Figure 3 shows the distributions of the capacity share on a route per aircraft type for the year 2016. The second ordinate displays the number of used aircraft units per aircraft type. The results show that the model overestimates the route capacity share of most aircraft types; especially for those with large numbers of used units and ASKs (see also Fig. 2), e.g., A320ceo, A330, B737-800 and B777.

**Fig. 4:**
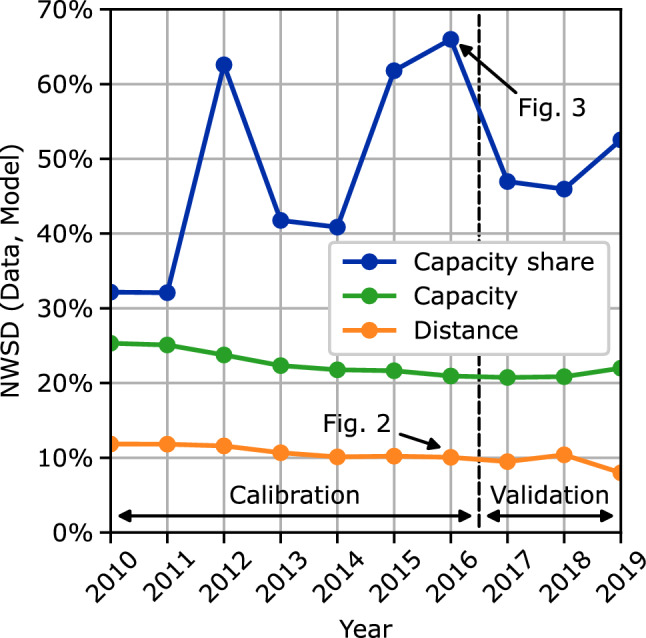
Comparison of normalized Wasserstein distances between historic flight schedule data and model results for the years 2010–2019

To quantify and summarize the previously described differences of the fleet assignments for multiple years and evaluation parameters, a normalized first Wasserstein distance (NWSD) approach is used. For each year the data and model distributions of each aircraft type are compared using the Wasserstein distance and normalized with the mean of the input data. The results of the different aircraft types are then ASK-weighted and averaged for each year. Figure 4 shows the results of the NWSD evaluation for the time span 2010–2019 for distance, capacity and capacity share distributions. The results show the lowest NWSD values for the distance distributions and the largest for the capacity share. Additionally, the results for the capacity share are also more volatile than for the distance and the capacity. Regarding the different time spans of calibration and validation, the NWSD results show no distinct differences; with the calibration including the data sets for 2010–2016, which were used for calculating the parameters $a_5$ through $a_{14}$ of the utilization submodel, the parameters $a_{25}$ and $a_{26}$ of the market concentration submodel and the weighting factors $\beta _{1}$ and $\beta _2$ of the objective function; and with the validation including the data sets for 2017–2019.

### Fleet assignment per route category

Aggregating and comparing the historic flight schedule fleet assignments and model results for routes with similar distance and capacity characteristics leads to the subsequently described results.

**Fig. 5:**
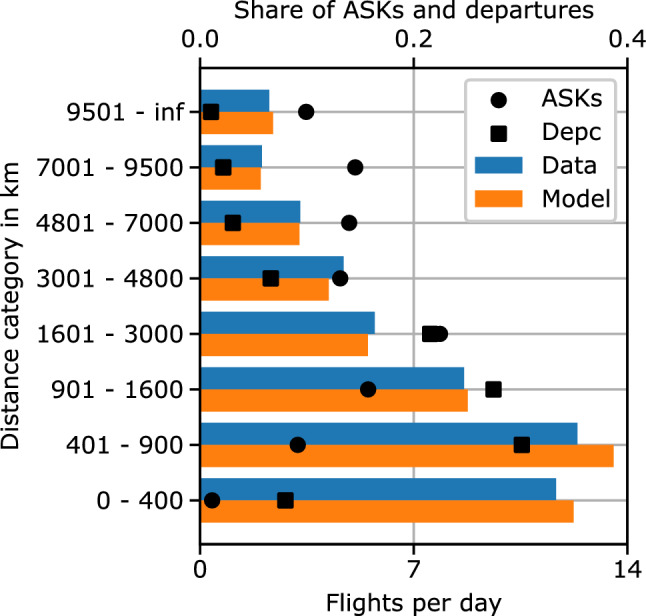
Comparison of historic flight schedule data with model results: average flight frequency in flights per day over distance categories for the year 2013

Figure 5 shows the average number of flights per day for routes in different distance categories for the year 2013. The importance of the different categories for the overall ATS is underlined by the relative ASK and departure count (Depc) shares of each category visualized on the upper abscissa. The results show smaller differences between input data and model results for categories with distances longer than 900 km and larger differences for shorter routes. However, the routes in the two shorter distance categories only represent about 10% of the overall ASKs.

**Fig. 6:**
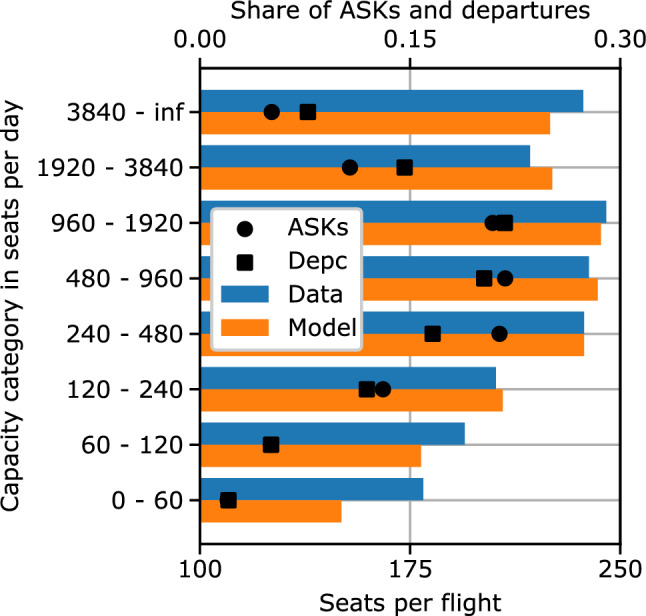
Comparison of historic flight schedule data with model results: average aircraft size in seats per flight over capacity categories for the year 2013

Figure 6 visualizes the average number of available seats per flight for different capacity categories for the year 2013. The results show the highest accuracy for capacity categories with more than 240 available seats per day. Together these categories represent about 90% of the ASKs and departures. The largest differences occur for low capacity routes with less than 120 available seats per day, representing about 10% of the ASKs and departures.

**Fig. 7:**
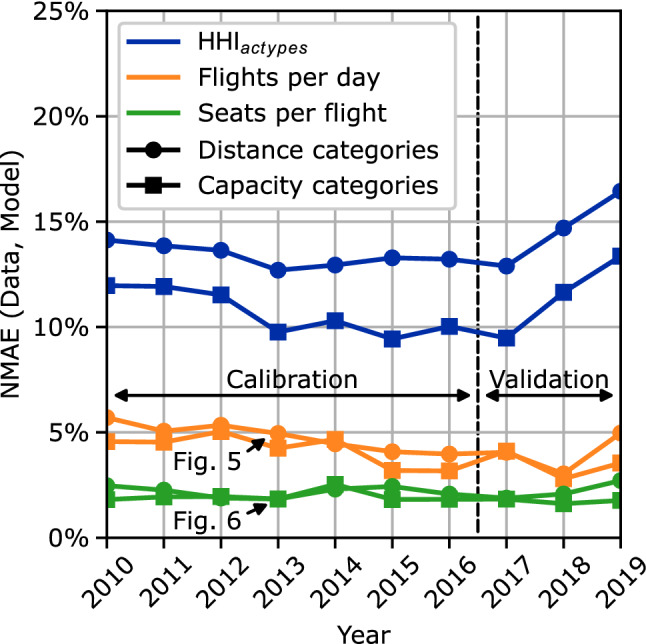
Normalized mean absolute error comparison between historic flight schedule data and model results for the years 2010–2019

Similar to the NWSD analysis performed around Fig. 4, the categorized results can also be quantified, summarized, and analyzed for different years and evaluation parameters. However, as the categorized results include only vectors of scalar values instead of vectors of discrete distributions, a normalized mean absolute error metric (NMAE) instead of the NWSD metric is used for data aggregation. Figure 7 shows the NMAE results for all combinations of distance and capacity categories with the evaluation parameters flights per day, seats per flight and HHI aircraft type market concentration per route. The results show the lowest error values for the seats per flight and the highest error values for the HHI-related evaluation parameters. For the validation time span, the HHI-related evaluation parameters show increasing error values, while the flights per day and seats per flight error values remain approximately constant.

### Airport capacity usage

**Fig. 8:**
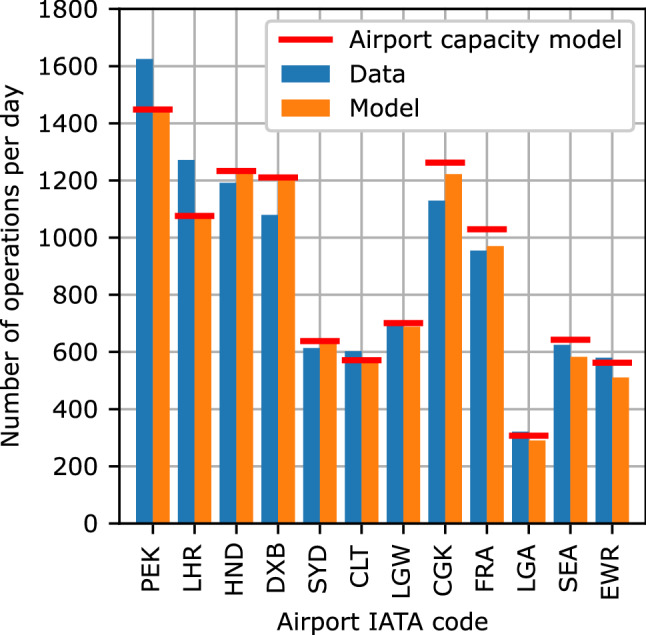
Number of runway operations per day for the twelve airports with the highest percentage usage of modeled airport capacity in 2016

Figure 8 shows the calculated runway capacity constraints and the number of runway operations per day for both the historic flight schedule data and the model results for the year 2016. The visualized data are used to check the accuracy of the airport capacity submodel as well as the local accuracy of the overall model. The depicted twelve airports are those with the highest relative runway capacity usage in the model results.

The comparison of the model results with the airport capacity submodel constraints indicates that only seven airports (PEK to LGW) are directly capacity constrained. For all other airports in the overall model the airport capacity submodel calculates a higher runway capacity than required. The capacities of four shown airports are overestimated (HND, DXB, CGK and FRA), two are underestimated (PEK and LHR) and the capacities of the other six shown airports are estimated with only minor differences. For the depicted airports, the largest difference between historic flight schedule data and model results, not caused by too restrictive constraints, occurs for Dubai International (DXB).

## Discussion

In the following, the previously described results are discussed and interpreted to draw conclusions for the overall model performance and potential future improvements.

First, both the aircraft type results (Figs. 2 and 3) and the categorized results (Figs. 5 and 6) show a higher accuracy of the overall model for aircraft types and route categories with high ASK shares. This is not surprising as most metrics within the model, including the calibration process, are ASK-weighted. Hence, the model focuses on minimizing the differences of high ASK categories and aircraft types. This behavior is expected to lead to the best overall results and is, thus, rated desirable for the global ATS scope of the overall model.

Additionally, low ASK categories and aircraft types are more sensitive to potential single outliers, caused by either route or airline characteristics. For instance, all B767-400 passenger aircraft included in the historic flight schedule data for 2016 are operated by two North American network carriers; an information surely affecting the resulting operational scenarios of this aircraft type, but unknown to, and out of scope of the model. Nevertheless, future work should include an in-depth analysis of the low ASK differences.

Second, the previously presented results show a relatively low accuracy of the overall model regarding the aircraft type capacity share and the aircraft type market concentration on individual routes, see Figs. 3, 4 and 7. Even though the market concentration submodel closely links the route capacity to an upper HHI limit, see Eq. (11), the resulting NMAE values regarding the HHI of the capacity categories are relatively large, see Fig. 7. Furthermore, the increasing NMAE of the HHI evaluation parameters during the validation time span might even indicate an overfit of the market concentration submodel to the calibration data. Additionally, the volatility in the NWSD capacity share results, see Fig. 4, may indicate a lacking significance of the model regarding the reproduction of the capacity share. In sum, the presented results underline that the current approach of using a market concentration submodel to include the effects of market segmentation and airline competition in the explanatory cross-airline approach needs to be rethought and improved. From a computational perspective, an alternative approach without the HHI would also be beneficial as calculating the HHI leads to the only quadratic constraints in the otherwise linear optimization problem, see Eq. (16).

Third, the exemplary airport results, see Fig. 8, indicate that a more precise airport capacity submodel could improve the accuracy of the overall model. Although the capacities of some airports are precisely reproduced or even underestimated, the capacities of the majority of airports are overestimated. While according to IATA information [53] a global total of 171 and 197 level 3 coordinated airports exist for the winter and summer season 2021, respectively, the model results only show seven airports operating at their capacity limit in 2016. Despite the five-year time difference of the comparison data, this might indicate that the airport capacity constraint in the overall model is currently too loose.

Additionally, the airport results for DXB underline the incapability of the presented modeling approach to reproduce local characteristics. As the hub of the largest A380 operator, Emirates, an accumulation of A380 flight operations occur at DXB. For the depicted year of 2016 a total of 47% of all A380 operations originate from, or terminate at DXB. As the global cross-airline model has no reason to assume such an accumulation of A380 operations in one airport, other smaller aircraft types are used for flights to and from DXB. Thence, the number of flight operations at DXB is overestimated, as seen in Fig. 8.

Finally and despite the above mentioned further improvement potential, the overall accuracy of the results shows that an explanatory approach to modeling the fleet assignment in the global ATS via an optimization problem is very much suitable. When reproducing the average flight frequency and aircraft size for different route categories the NMAEs are smaller than 6%. For the aircraft type distance distributions, the NWSDs between model results and historic flight schedule data are smaller than 12%.

In comparison to fleet assignment models in the literature for individual airlines or multiple competing airlines, we made additional assumptions and approximations to allow global fleet assignment modeling. The overall accuracy of the results shows that these assumptions and approximations are reasonable, given the global and more aggregated scope within this study.

In comparison to other global fleet assignment models available in the literature, e.g., AIM [25–27] and FoAM [28], our new explanatory modeling approach leads to two main advantages. First, the presented modeling approach allows for aircraft types with similar size but different operational capabilities. This is especially useful when analyzing future ATS scenarios, which include both conventional and new propulsion technology aircraft and the latter ones potentially having different range capabilities. For example, Wehrspohn et al. [54] use the model presented in this study to determine a potential operating scenario of a future hybrid-electric hydrogen aircraft concept, which has the same size as an older conventional large short-haul aircraft but a shorter design range. Second, the explanatory approach enables analyzing the influences of different future economic and operational scenarios as well as of different fleet compositions on the global fleet assignment. For instance, ATS scenarios with high energy cost will lead to more energy efficient aircraft being deployed on long-range routes, while less efficient aircraft are used on shorter range operations. Likewise, ATS scenarios with limited airport capacities in one region and more available capacities in other regions will lead to the utilization of larger aircraft in the first region and smaller aircraft in the others. Additionally, ATS scenarios with different fleet compositions—especially if these compositions include new aircraft sizes or exclude all aircraft of similar size—will lead to changes in the fleet assignment, which the statistical approaches of FoAM and AIM cannot model.

Within this study, the global aircraft fleet and the global route network with its offered passenger seat capacity per route are constant inputs generated from historic data. Thus, the interactions of passenger demand, fleet assignment, and fleet development are not within the scope of this study. However, for other studies, like the introduction of a new aircraft type in the future [55], this can be changed by integrating the presented model into a simulation framework with feedback loops to distinct models for passenger demand and preferences [56] as well as fleet development.

When using the presented model for future scenarios, this is done under the assumption that the calibration of the submodels and objective function with data from the years 2010–2016 remains valid. This implies that the overall structure of the ATS remains comparable to the calibration time span, e.g., regarding the competition of airlines, the number of available aircraft types and the balance of demand and supply. While there currently is no inherent reason to assume differently, a recalibration to newer data might become necessary in the future. In addition, a sensitivity analysis regarding the calibration time span could give insights into the uncertainty for future scenarios.

## Conclusion

This article presents a new methodology for modeling the fleet assignment in the global ATS. The new methodology is based on an explanatory cross-airline approach; formulating and solving an optimization problem representing the fleet assignment in the global ATS. The objective function of this optimization problem includes the airline perspective via operating cost and the passenger perspective via schedule delay and travel time. The optimization constraints include logical, operational, and technological aspects of the fleet assignment, like payload-range and airport capacity constraints.

The presented results demonstrate the overall suitability of this approach, while also indicating improvement potential for future work. When reproducing the fleet assignment of 35 different aircraft types for the years 2010–2019, the results show errors of less than 6% for the average route flight frequency and aircraft size in comparison to historic flight schedule data. Similarly, the distance distributions of individual aircraft types are reproduced with errors of less than 12%. Larger errors occur for the aircraft type market concentration and capacity share.

The accuracy of the overall model can most likely be increased by improving the existing objective function and constraints of the optimization problem. Alternatively, new constraints and additional terms to the objective function should be investigated. Additional aircraft types, in particular regional and commuter aircraft, can improve the applicability of the presented model. Moreover, future work should focus on the application of the presented model. Potential research questions include modeling and analyzing the fleet assignment for future global ATS scenarios as well as deriving operational scenarios for future (green aviation) aircraft designs.
